# Complement in metabolic disease: metaflammation and a two-edged sword

**DOI:** 10.1007/s00281-021-00873-w

**Published:** 2021-06-22

**Authors:** B. C. King, A. M. Blom

**Affiliations:** Department of Translational Medicine, Lund University, Lund, Sweden

**Keywords:** Complement, Diabetes, Obesity, Inflammation, Adipocyte, Insulin, CD59, C3, C4BP

## Abstract

We are currently experiencing an enduring global epidemic of obesity and diabetes. It is now understood that chronic low-grade tissue inflammation plays an important role in metabolic disease, brought upon by increased uptake of a so-called Western diet, and a more sedentary lifestyle. Many evolutionarily conserved links exist between metabolism and the immune system, and an imbalance in this system induced by chronic over-nutrition has been termed ‘metaflammation’. The complement system is an important and evolutionarily ancient part of innate immunity, but recent work has revealed that complement not only is involved in the recognition of pathogens and induction of inflammation, but also plays important roles in cellular and tissue homeostasis. Complement can therefore contribute both positively and negatively to metabolic control, depending on the nature and anatomical site of its activity. This review will therefore focus on the interactions of complement with mechanisms and tissues relevant for metabolic control, obesity and diabetes.

## The complement system

The complement system is an evolutionarily ancient mechanism of humoral innate immunity, composed of many serum proteins that activate in a sequential chain reaction, raising an immunological alarm upon pathogen detection (1). Activation products of complement are able to signal to and activate immune and endothelial cells, label pathogens for enhanced uptake and destruction by phagocytes and also kill some pathogens directly, by disruption of their surface membranes.

**Fig. 1:**
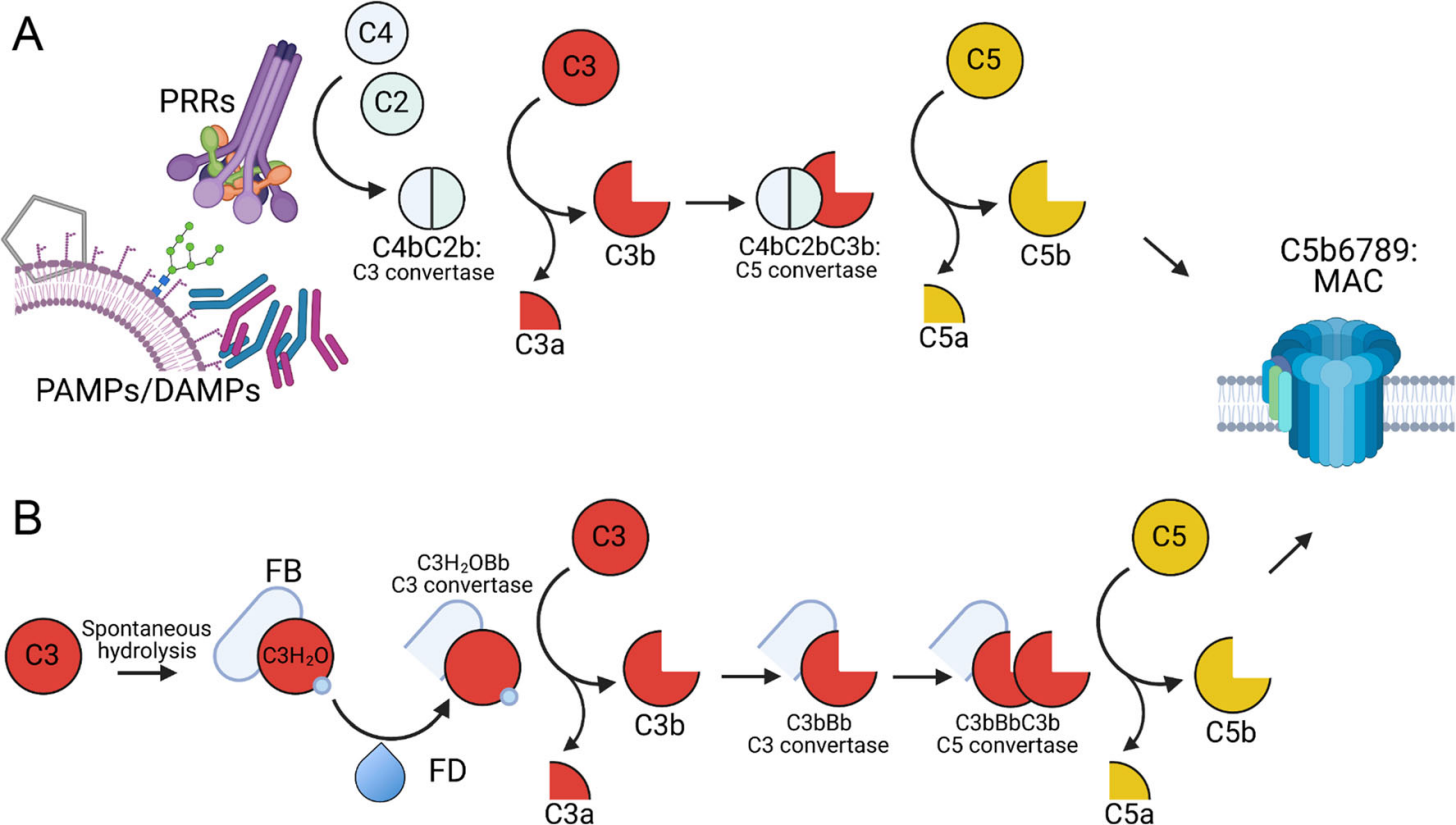
Pathways of complement activation. **A** The classical and lectin pathways are activated by the PRRs, C1Q and MBL/ficolins respectively, which recognise PAMPs and DAMPS such as bound antibodies, dead cells and foreign or altered carbohydrates. PRR-associated proteases cleave C2 and C4, which form the C3 convertase, and subsequently cleave C3 into C3b and the anaphylatoxin C3a. C3b itself associates with C4bC2b to form the C5 convertase, which cleaves C5 into the potent anaphylatoxin C5a, and C5b. C5b then associates with complement components 6-9. Poly-C9 forms a membrane-breaching pore that can directly lyse gram-negative bacteria. **B** The alternative pathway is initiated by spontaneous hydrolysis of C3 to C3H_2_O, to which FB can bind. Subsequent conformational changes allow serum protease FD cleave FB to Bb, and C3H_2_OBb is the initial alternative pathway C3 convertase. FB also forms a convertase with subsequent C3b products, causing amplification unless regulated by FH. Incorporation of C3b into the C3 convertase allows cleavage of C5, ultimately leading to MAC formation. The alternative pathway is also involved in amplification of the classical and lectin pathways. Previously, C2b was referred to as C2a (2). For further details, see text

There are three canonical activation pathways of complement. For two of these pathways, soluble pathogen- or danger-sensing molecules of complement (pattern recognition receptors, PRRs) can directly recognise pathogen- or danger-associated molecular patterns (PAMPs, DAMPs) (Fig. 1A). The first of these two pathways is the lectin pathway, whereby mannose-binding lectin (MBL), or one of several ficolins, bind to and recognise foreign or altered carbohydrate groups on pathogen surfaces or glycoproteins (3). The second is the classical pathway, so named because it was the first identified complement pathway, whereby the PRR C1q binds to antibody conformations arranged on surfaces of an antigen-positive target (4). Upon binding of these receptors, conformational changes occur and associated proteases become activated (MBL-associated serine proteases, MASPs, for the lectin pathway, or C1s and C1r associated with C1q in the classical pathway), cleaving the first components of complement activation, complement components 2 and 4 (C2, C4). The resultant cleavage products, C4b and C2b, together form an enzymatic complex covalently anchored to the activating surface, the C3 convertase, which cleaves complement component 3 (C3), which is the central or ‘hub’ component of the complement system.

C3 is a fascinating and multifunctional protein (5). When cleaved by its convertase, a peptide, C3a, is released, resulting in a large conformational change in the remaining protein, now called C3b (6). An ‘arm’ of C3b unfolds towards the activating surface, revealing a highly reactive thioester group within the thioester domain (TED), an unusual but highly conserved domain that functions in immune defence across the animal kingdom, from sponges (7) and corals (8), to insects and mammals. Once revealed, the thioester group reacts rapidly with adjacent hydroxy or amine groups, forming a covalent bond and irreversibly anchoring C3b to the surface via the thioester domain. In this way, the first function of complement is achieved, labelling pathogens with C3 activation products, which are themselves recognised by specific complement receptors on phagocytic cells, enhancing uptake and killing of pathogens.

Once C3b is deposited on activating surfaces, it can also interact with the C3 convertase, forming a larger complex with higher affinity for the next complement component, the serum protein C5. Cleavage of C5 also releases a peptide, C5a, and induces conformational change in the product, C5b. C5b can bind to additional serum complement proteins C6 and C7, forming a complex, which then inserts into the plasma membrane of cells or gram-negative bacteria. This allows recruitment of serum complement component C8, and multiple copies of C9, which insert into the membrane and form a ring-like membrane-breaching pore, the membrane attach complex (MAC), capable of inducing rapid killing of target bacteria and cells, as membrane integrity is lost and cellular contents escape(9). This terminal activation pathway of complement represents a second canonical function of complement, direct killing of target cells.

The third important canonical function of complement is the production of the anaphylatoxins. Cleavage of C4, C3 and C5 all lead to release of peptides, C4a, C3a and C5a, of which C3a and C5a are considered the most pro-inflammatory. These bind specific receptors on both immune and non-immune cells, causing contraction of smooth muscle cells, vasodilation, degranulation of mast cells, cellular activation and chemotaxis of leukocytes to the site of complement activation, many of the signs of acute inflammation. Indeed, the symptoms of anaphylaxis are what give these peptides their group name of the anaphylatoxins. Complement is therefore a multifunctional system, capable of sensing danger via PRRs, and translating these danger signals into a whole range of appropriate acute responses, both activating and attracting effector cells, covalently labelling targets for uptake and clearance, and also causing direct destruction of some pathogens via MAC, acting on a timescale of minutes. Longer-term, complement activation also interacts with and acts as adjuvant to activation of the slower adaptive immune system, and generation of antigen-specific responses, a subject well-covered elsewhere (10).

As well as the classical and lectin pathways, a third pathway of complement activation exists, the alternative pathway (Fig. 1B). This was the last to be discovered but is the most evolutionarily ancient. While the classical pathway evolved relatively recently with the development of adaptive immunity, components of the alternative pathway evolved first, with both C3 and complement factor B (FB) having been identified in *Oscarella carmella*, a species of sponge (7), representing nearly the most evolutionarily ancient stage of multicellular animal life. The alternative pathway relies on the slow tick-over-activation that occurs by spontaneous hydrolysis of C3, whereby the thioester domain, although protected within the structure of intact C3, reacts with H_2_O to form C3H_2_O, which then undergoes a conformational change similar to C3b. Although the thioester group is neutralised and opsonisation cannot occur, C3H_2_O now binds serum protein FB, which is then cleaved by complement factor D (FD), also called adipsin. The complex of C3H_2_OBb is now a soluble C3 convertase and can cleave further copies of C3 into C3b. The spontaneous alternative pathway-mediated turnover of C3 is significant, at about 1% of C3 every hour (11), and is regulated by soluble complement inhibitors, the most important of which is factor H (FH). FH dissociates the convertase and causes inactivation of C3H_2_O by acting as a cofactor to serum protease factor I, which cleaves C3H_2_O/C3b. The activating potential of the alternative pathway can be seen in knockout mice lacking FH; in these animals, the alternative pathway runs out of control, producing more and more copies of C3 convertase until the entire serum content of C3 is consumed, resulting in extremely low levels of serum C3, but high deposition of complement activation products, tissue damage and loss of function in vulnerable tissues, such as the kidneys (12) and eyes (13). Consequently, human FH polymorphisms that reduce its inhibitory activity are also associated with complement-mediated kidney and eye disease (14, 15).

The complement system is therefore both a signalling and effector mechanism involved in innate immune responses, but also has important roles in homeostasis and clearance of unwanted self-material, such as immune aggregates and apoptotic debris (16). Recent work has revealed that local complement activation and signalling can also function in other systems, particularly in neural development (17), and the proposal of autocrine functions of intracellular complement proteins has lead to speculation as to the very origin of complement, with potential evolutionary links to cellular metabolic pathways (18). This review will highlight the interactions between the complement system and major tissues involved in metabolic disease, with focus on the current epidemics of obesity and diabetes.

## Diabetes

Diabetes is a major global human health burden, with hundreds of millions already currently affected worldwide, and with most rapid increases in incidence in developing countries. Diabetes is defined by a loss of control of blood glucose regulation, caused by an inability of insulin secretion to sufficiently meet the demand to clear blood glucose. As shall be described, this can be due to compromised levels of insulin secretion, a resistance of target tissues to respond to insulin, or a combination of both. An insufficiency of insulin relative to need leads to hyperglycaemia and resultant complications, including peripheral neuropathy, retinopathy and nephropathy.

Blood glucose homeostasis is reliant on the interplay of many different tissues, which must be understood in order to explain the pathogenesis of diabetes. In the healthy individual, breakdown of carbohydrates in food and subsequent absorption leads to an increase in blood glucose levels, which is quickly detected by β-cells found within the islets of Langerhans in the pancreas. Although making up only about 1% of the total pancreatic mass, the islets receive about 10–20% of its total blood flow (19), being exceptionally well-vascularised. Expression of high-capacity glucose transporters leads to rapid uptake of glucose into β-cells, which is rapidly metabolised by mitochondrial oxidative phosphorylation, altering the intracellular ATP/ADP ratio. This leads to the opening of cell-surface ATP-sensitive potassium channels, causing membrane depolarisation and activation of voltage-gated calcium channels. This allows calcium entry into the cell, activating the calcium-dependent machinery of the exocytotic pathway, and ultimately leading to fusion of secretory granules with the cell surface, and release of insulin to the extracellular environment. Insulin acts on target tissues such as skeletal muscle and liver, triggering trafficking of intracellular pools of glucose transporters to the cell surface and rapid clearance of glucose from the blood for intracellular storage as glycogen. Insulin also has important effects on adipose tissue and lipid storage, decreasing rates of lipolysis and instead inducing triglyceride uptake (20). Diabetic patients with a relative lack of insulin production therefore often have increased plasma levels of both glucose and triglycerides.

Diabetes has historically been divided into two subsets, type 1 and type 2. Type 1 diabetes (T1D) is an autoimmune disease typically defined by presence of autoantibodies against pancreatic islet antigens, involving an adaptive immune response-mediated destruction of pancreatic β-cells, destroying the source of insulin secretion. T1D typically has an early age of onset, and like many other autoimmune diseases, is linked to the HLA region of the genome, which defines which peptide antigens the adaptive immune system can recognise and therefore react against. The non-obese diabetic (NOD) mouse is a classic mouse model of T1D, which develop autoimmune T-cell-dependent diabetes at an early age. These mice were found to lack functional C5 (21), ruling out the involvement of the terminal pathways of complement in this model. Nevertheless, the presence of complement-fixing anti-islet antibodies has been demonstrated in sera from human T1D patients (22, 23), demonstrating a potential role of complement-mediated β-cell apoptosis and islet inflammation in the human disease. The development of autoimmune diabetes in a streptozotocin-induced mouse model was also found to be dependent on expression of C3 within immune cells (24), most likely reflective of recent discoveries of autocrine functions of complement in immune cell activation and survival (25).

Type 2 diabetes (T2D), which makes up at least 90% of new diabetes diagnoses, has been linked to obesity, ageing and a so-called Western lifestyle, and is the most rapidly increasing form of the disease. T2D may develop due to a lack of insulin secretion, a loss of sensitivity of the target tissue to insulin signalling (known as insulin resistance), or a combination of both. This paradigm has been soundly validated by recent large-scale studies of thousands of human diabetes patients in various geographic locations (26–28), which have further stratified T2D into several subsets (29) (Table 1). These studies took into account measurements of β-cell function and insulin secretion, insulin sensitivity, BMI, age of onset, glycated haemoglobin and presence of autoantibodies, and identified 4 subtypes of T2D in addition to T1D. Of note, severe insulin-deficient diabetes (SIDD) and severe insulin resistance diabetes (SIRS) are two separately defined subtypes, with differing genetic associations(26, 29), showing that separate pathogenic mechanisms in different tissues may be responsible for producing different subsets of T2D.

**Table 1:** New definitions of clinical subtypes of diabetes, demonstrating heterogeneity of pathology (simplified from reference (26))

Subtype	%	Autoantibodies	Insulin secretion	Insulin resistance	BMI	Particular risks
Severe autoimmune diabetes (SAID)	6	+++	-	-	+	-
Severe insulin-deficient diabetes (SIDD)	18	-	-	+	+	Retinopathy, neuropathy
Severe insulin-resistant diabetes (SIRD)	15	-	++	++	++	Nephropathy fatty liver
Mild obesity-related diabetes (MOD)	22	-	+	+	+++	-
Mild age-related diabetes (MARD)	39	-	+	-	+	Low risks

Importance of individual disease features were scored from -, (unimportant), to +++ (highly important) to show the relative incidence or importance of these features to the different subtypes of diabetes

## Evidence for complement involvement in diabetic complications

Development of clinical diabetes and subsequent prolonged elevated blood glucose levels leads to not only known complications, including neuropathy, but also renal and retinal diseases. Indeed, deposition of complement activation products is a feature of both diabetes-related retinopathy (30) and nephropathy (31), although there is also evidence for lectin and classical pathway involvement; serum MBL levels are a strong biomarker for diabetic nephropathy in both T1D and T2D, and C4b as well as C1q were found deposited in human kidney samples from diabetic patients, correlating with nephropathy (32).

There is evidence that raised blood glucose levels can lead directly to dysregulation of complement inhibition, therefore leading to activation of complement and direct tissue pathology. Under prolonged exposure, plasma glucose can react with cell-surface molecules, chemically modifying them via a glycation reaction. Advanced glycation end products can directly activate complement via altered recognition by the carbohydrate-sensing PRR MBL (3), leading to complement deposition on endothelial surfaces. In addition, glycation can also inhibit protein function, for example in the case of CD59, a ubiquitously expressed inhibitor of MAC assembly. Under prolonged exposure to increased glucose, CD59 becomes glycated at its C5b-8 binding site, therefore inactivating CD59 and allowing increased complement MAC formation at the cell surface (33). Due to the specificity of this glycation reaction and its requirement for prolonged elevated blood glucose levels, glycated CD59 has been assessed as a promising novel biomarker for gestational diabetes (34). Specific monoclonal antibodies detected glycated CD59 colocalising with MAC in kidneys and nerves from diabetic but not non-diabetic subjects (35), implicating glycation-mediated CD59 inactivation in complement-mediated diabetes-related nephropathy and neuropathy. Intense MAC staining has also been found in choriocapillaris of the eyes of diabetic retinopathy patients (30).

Diabetes is therefore associated with organ damage in eyes, kidneys and nerves, all tissues known to be sensitive to complement attack, as demonstrated by significantly increased risks of pathology at these sites in individuals harbouring polymorphisms or mutations in complement inhibitors (36), independent of diabetes. Deposition of activated complement proteins at these sites in diabetic patients, together with evidence for explanatory complement-activating mechanisms and CD59 inactivation, point to dysregulation of the complement system in diabetic complications. In support of this hypothesis, a recent study also found that a FH polymorphism significantly lowering plasma FH levels increased the risk for both renal dysfunction and cardiovascular events in a study of over 1100 human T2D patients (37).

## The complement system in adipose tissue

T2D is often associated with obesity, although as the recent T2D reclassifications show, it is possible to be overweight and metabolically healthy, as well as lean but diabetic. Obesity is associated with increased circulating markers of inflammation (38, 39), including the acute phase complement proteins such as C3 (40); circulating C3 levels were found to be predictive of future diabetes development in large patient cohorts (41, 42). Key studies in both obese people and mice also show that there are substantial changes in the populations of immune cells found in adipose tissue during obesity (43–46), with a skew towards pro-inflammatory phenotypes. Adipose tissue is not simply a passive site of lipid storage, but is an important endocrine tissue central to nutrient homeostasis (47), and a source of secreted adipokines that influence the brain, liver, muscle, gonads, vasculature and lymphoid organs (48). The normal function of adipose tissue is altered by obesity-induced inflammation, and low-grade tissue inflammation is now seen as an important factor in obesity-associated insulin resistance, leading to T2D development (49). There is therefore an interaction between adipose tissue and the innate immune system, which becomes altered in obesity, and which impacts upon metabolic function of adipocytes.

Adipose tissue is of central importance to the complement system, as FD, an essential component of the alternative pathway of complement activation, is produced primarily in adipocytes, where it was originally named adipsin before these two factors were discovered to be identical (50). FD cleaves FB into Bb and is therefore essential for activation of the alternative pathway of complement. Adiponectin-deficient mice completely lacking adipose tissue are also completely deficient in circulating FD, with minimal alternative pathway activity, demonstrating that adipose tissue is the primary source of circulating FD in rodents (51). FD is expressed as a zymogen, and the complement protein MASP-3 has been identified as the key factor that processes pro-FD into its active form (52). The MASP-3 protein is an alternative splice product from the MASP1 gene, and MASP1/3 knockout mice therefore have no functional FD, and subsequently a defective alternative pathway (53). These mice were also reported to weigh less than littermate controls, and had adipocytes of smaller size, suggesting defects in adipocyte development or lipid storage (54), demonstrating functional links between adipose tissue and the alternative pathway of complement activation.

This link has been attributed by one research group to inactivated C3a; once cleaved from C3 during complement activation, C3a can be rapidly inactivated by removal of a single C-terminal arginine residue, forming C3a-desArg. Baldo et al. identified that C3a-desArg stimulated lipogenesis in adipocytes (55), the synthesis of triglycerides for storage, and the same group also found that aged female C3-knockout mice, which therefore also lack C3a, were resistant to increases in body weight on a high-fat diet, and had lower blood levels of glucose and insulin (56), signifying improved metabolic homeostasis. In addition, these C3-KO mice, similar to MASP1/3 KO mice (54), had lower circulating levels of the appetite-regulating hormone leptin, which is also secreted from adipocytes. These results have however proven controversial, partly because of the fact that the interaction of C3a-DesArg with the proposed receptor, C5L2 (57), has been disproven by other groups (58, 59). In addition, reproduction studies failed to confirm any changes in serum levels of free fatty acids, cholesterol or triglycerides between C3-KO or WT mice (60), although this was proposed to be due to differences in mouse genetic background (61), something that can dramatically affect mouse nutrient homeostasis and metabolism (62). The use of total C3-KO mice in these studies to specifically study the role of C3a-DesArg in adipocytes is also far from optimal, given the many other known and potential roles of C3 in other tissue types. However, pharmacological blockade of the complement anaphylatoxin receptors C3aR and C5aR in rats was shown to reverse high-fat diet-induced visceral adiposity and both glucose and insulin intolerance (63).

In addition to FD, adipocytes also express and secrete C3 and FB, all the necessary components for alternative pathway activation. These are upregulated in adipocytes from both mice and human donors during differentiation (64) and after stimulation with pro-inflammatory cytokines (65), during which the alternative pathway becomes activated, with production of cleaved activation products of both FB and C3, showing that an autocrine alternative pathway can be activated by adipocytes, potentially forming a pro-inflammatory feedback loop (66). In fact, it has been shown that activation of complement, and production of anaphylatoxins, in particular, is responsible for induction of adipose tissue inflammation under high-fat diet feeding (63) and recruitment of pro-inflammatory macrophages into adipose tissue in diabetic mouse models (65, 67). In a recent extensive meta-analysis of genome-wide association studies analysing millions of single nucleotide variants in over 150,000 individuals, regulatory enhancer analysis revealed that genetic loci associated with insulin resistance showed tissue-specific enrichment for macrophages, strongly implicating this cell type in insulin resistance (68). The production of pro-inflammatory cytokines by these macrophages actually induces insulin resistance in adipocytes, rendering them less sensitive to insulin signalling (69), which leads to impaired blood glucose clearance, and compensatory increases in β-cell insulin expression, which contributes to ER stress and β-cell exhaustion. Evidence therefore points to local adipose tissue complement factor expression and activation contributing to adipose inflammation, recruitment of pro-inflammatory cells and subsequent induction of insulin resistance.

Serum samples from human lipodystrophy patients show a linear relationship between adipose mass and circulating FD levels. Accordingly, levels of FD in human patients correlate with both BMI and waist circumference (70), consistent with adipose tissue being the main known source of serum FD. As obesity is a clear risk factor for development of T2D and is linked to inflammation, and inflammatory cytokines stimulate increased adipocyte FD expression, it may therefore be expected that an increase in adiposity, and therefore an increase in FD, would correlate with diabetes development. However, the picture is more complex than this; not all adipose tissue is equal. Circulating FD does not originate equally from adipose tissue at different anatomical sites; high expression of FD is linked to subcutaneous, but not visceral fat (70), and visceral fat specifically, is a risk factor for T2D development, while subcutaneous fat may even be protective (71); transplanting subcutaneous fat into the visceral compartment can even lead to reduced overall adiposity and improved glucose homeostasis (72). While overall increased BMI is a risk factor for T2D development, increased FD levels are correlated with decreased T2D incidence, and improved fasting blood glucose levels (70). In fact, diabetic and obese individuals, and even obese rodent models of T2D, have significantly lower serum levels of FD (50, 73–75), while mRNA levels of FD are increased in normal rats during fasting (73). While FD is therefore derived primarily from adipocytes, there are therefore clear differences in the function of adipose tissue at different anatomical sites, with differing associations between anatomical site, FD production, and contribution to T2D development. It should also be noted that circulating FD levels relate to renal function, as at only 24 kDa, FD is cleared from the blood by glomerular filtration (76), which is initially increased in hyperglycaemia, but can be affected by diabetes-associated kidney disease.

Evidence of the differing complement activity of adipose tissue at anatomical different sites can also be found in cases of acquired partial lipodystrophy (APL). APL is a disorder that leads to loss of adipose tissue from specific anatomical sites, typically from the upper body (the face, arms and abdomen), but not from the buttocks or legs, leading to a wildly skewed fat distribution. Loss of adipose storage depots leads instead to ectopic lipid deposition in muscle and liver, leading to fatty liver disease and liver failure. Increased circulating triglyceride levels also contribute to insulin resistance of other tissues. APL is currently thought to be largely complement-mediated, with 83% of patients in one study testing positive for nephritic factor, an autoantibody that stabilises the alternative pathway C3 convertase C3bBb, leading to complement over-activation and consumption (77), which presents as very low patient serum C3 levels. APL patients were also at a highly increased risk of developing a complement-mediated kidney disease (78), membranoproliferative glomerulonephritis (MPGN). The question therefore arises as to why only certain anatomical sites in these patients are vulnerable to loss of adipocytes in the presence of nephritic factor. Adipocytes themselves express all the necessary components for alternative pathway activation, and so it could be speculated that nephritic factor stabilises this convertase at the site where it is most efficiently produced, leading to over-activation of complement and inducing death of adipocytes at those sites. This hypothesis is supported by in vitro induction of adipocyte apoptosis by nephritic factor (79). This would therefore indicate that adipocytes at the sites most vulnerable to nephritic factor are also those producing highest amounts of the alternative pathway components, particularly FD, therefore suggesting that adipose tissue in the upper body likely expresses higher amounts of FD. Inflammation induced by nephritic factor could also induce further expression of complement components by adipocytes at these sites (66), leading to positive feedback of complement activation and expression, and resultant pathology.

## The control centre: functions of complement in pancreatic islets

The main function of pancreatic islets is the regulation of blood glucose levels by secretion of β-cell-derived insulin, and α-cell-derived glucagon. The pancreatic islet is therefore the control centre of blood glucose regulation. Diabetes is caused by a relative deficiency of insulin compared to need (80), which can be caused by loss of β-cell and insulin secretion capacity, or decreased effectiveness of insulin due to development of insulin resistance in target tissues, or a combination of the two. While immune infiltration of the pancreatic islet, autoimmune attack and β-cell loss are the hallmarks of T1D, infiltration of pro-inflammatory cells and associated loss of function also occurs in T2D islet (81), as also witnessed in rodent models of diet-induced diabetes (82). While the pro-inflammatory potential of the complement system is well understood, investigations into the role of complement in the pancreatic islet have led to surprising findings as to non-canonical, homeostatic and protective functions of individual complement proteins.

## C3/C3a

As described above, cellular metabolism of β-cells, and the subsequent intracellular ATP/ADP ratio, directly influences insulin secretion. In recent years, it has been discovered that the activation of complement can act as a switch for cellular metabolism. Once activated, C3b acts as a ligand for the complement inhibitor and cell-surface receptor CD46, and CD46 ligation leads to potent activation of human CD4^+^ T-cells (83). T-cell activation and subsequent differentiation involve induction of rapid proliferation and upregulation of metabolism, and this was found to be dependent on CD46 signalling (84, 85). Complement activation products can therefore have direct effects on cellular metabolism on shorter timescales. Similarly, the C3 activation product, C3a, has been shown to stimulate β-cell metabolism, causing increases in oxidative phosphorylation (75). Consequently, the resultant increase in ATP/ADP ratio also meant that C3a augments insulin secretion. C3a can be produced by the alternative pathway convertase, formed when FB binds to C3b/hydrolysed C3H_2_O, and is cleaved by FD. Although FD/adipsin is decreased in sera of obese/diabetic humans and rodents, replenishing serum FD levels by viral re-introduction into obese diabetic ob/ob mice (75) rescued blood glucose homeostasis. This was attributed to the recovery of the alternative pathway function, and production of C3a, which presumably acts on β-cell. In a follow-up paper, it was also shown that C3a blocked β-cell de-differentiation and inhibited apoptosis, via regulation of phosphatase DUSP6 (70). These results together suggest a profound effect of the alternative pathway and C3a in particular on β-cell identity, function and survival. The fact that adipocytes are the main source of FD means that the complement system is a means of communication between adipose tissue and pancreatic islets, and regulation of expression of these factors at these different sites is therefore of direct relevance to metabolic control.

C3 is highly expressed in isolated human pancreatic islets (86), and expression analysis revealed that C3 expression is significantly upregulated in islets from T2D patients compared to healthy donors, and is upregulated in islets in multiple rodent models of diabetes (87). C3 secretion from isolated human islets is augmented by IL-1β exposure, and C3 expression in freshly isolated islets correlated with expression of pro-inflammatory cytokines, as well as with donor body mass index, and HbA1c, linking islet C3 expression with islet inflammation, obesity and diabetes. Surprisingly, protein interaction microarrays and ELISA confirmation revealed an interaction with isoforms of ATG16L1, a protein central to autophagy, and consequently, CRISPR/Cas9-mediated knockout of the C3 gene in β-cell INS-1 832/13 clones led to dysfunctional autophagy, whereby autophagosomes were unable to mature, and accumulated within the cell. The failure of autophagy in these cells led to increased apoptosis under exposure to stress-inducing diabetogenic factors, such as exposure to glucolipotoxic conditions, or IAPP (87). Isolated islets from C3 knockout mice also displayed a dysfunctional autophagic phenotype. These findings strongly implicate a non-canonical role for C3 in regulating autophagy within β-cells.

ATG16L1 is found within the cytosol, whereas C3 is canonically found in the secretory pathway and extracellular environment. The interaction of C3 with ATG16L1 has been demonstrated within the cytosol in the context of pathogen invasion, whereby C3-opsonised bacteria entering the cytosol are detected by ATG16L1 and targeted by autophagy (88). However, results indicating that a direct interaction between C3 and ATG16L1 regulates homeostatic autophagy pose a challenge regarding the subcellular localisation of these proteins. A solution was found by identification of in-frame alternative start codons found within C3 cDNA, directly downstream of the signal peptide. Use of these codons would result in C3 protein expressed directly into the cytosol. This was confirmed by mutation of the canonical ATG start site, whereby significant levels of C3 were expressed within the cytosol of cells, with no C3 secretion (87). Although activation products of canonical complement activation pathways have been shown to regulate autophagy via ligation of cell-surface receptors (89), the interaction of C3 directly with ATG16L1 represents a new mode in which intracellular, cytosolic isoforms of C3 promote pro-survival homeostatic cellular processes (90).

Islet-expressed C3 has also been shown to be a pro-survival factor during inflammation, with siRNA-mediated C3 knockdown leading to increased apoptosis in dispersed islet cells exposed to pro-inflammatory cytokines (91). Here, unlike for autophagy dysfunction, exogenously added C3 partially rescued the effect of C3 knockdown, and C3aR was implicated in mediating the effect. In lung epithelial cells, intracellular stores of C3 have also been shown to mediate cytoprotection against oxidative stress (92), and ‘stores’ of intracellular C3 have also been implicated in CD4^+^ T-cell survival (25). C3, and possibly C3a, may therefore have several roles within the islet, regulating autophagy, insulin secretion and β-cell identity, but also promoting cellular survival during stress and inflammation. The comparative contributions of exogenous or secreted C3 compared to intracellular C3 remains to be investigated, as well as the balance between these novel protective effects of C3, compared to potential deleterious outcomes of canonical pro-inflammatory extracellular complement activation within the islet.

## CD59

As described above, the terminal pathway of complement activation leads to production of the pore-forming MAC, which deposits into membranes. In order for host cells to protect themselves from MAC-dependent membrane damage, all human cells express CD59, a small protein that is anchored to the cell surface by a glycosylphosphatidylinositol (GPI) anchor. The GPI anchor consists of glycolipid post-translational modification that is added to the nascent protein within the endoplasmic reticulum, before transport to the cell surface. CD59 is able to intercept C5b67 after it inserts into the cell membrane, but before C9 is recruited to form the membrane-breaching pore. By blocking C9 integration, CD59 therefore prevents MAC formation and subsequent cell damage. The importance of this is seen in patients that experience somatic mutations in haematopoeitic stem cells, in the PIG-A gene, which encodes an enzyme required for GPI anchor synthesis. The resultant CD59-deficient red blood cells are susceptible to complement-mediated lysis, a disease known as paroxysmal nocturnal haemoglobinuria (93).

During investigation of how complement proteins may contribute to β-cell function, we knocked down CD59 in β-cell clones using siRNA, and discovered that cells lacking CD59 expression were unable to secrete insulin in response to glucose (86). Knockdown cells were also unresponsive to high potassium, which ‘short-circuits’ insulin secretion signalling by causing membrane potential depolarisation, suggesting that CD59 functions towards the distal end of the insulin secretion pathway. Proximity ligation assays also showed a co-localisation of CD59 with syntaxin and VAMP2, proteins located to the cytosolic facing membranes of the cell surface and insulin granules respectively, again suggesting that CD59 is involved in the mechanics of insulin granule fusion with the cell surface. Furthermore, in contrast to results with siRNA, enzyme-mediated removal of CD59 from the cell surface by cleavage of GP anchors did not affect insulin secretion, suggesting that the pool of CD59 involved in GSIS resides within the cell. These results therefore revealed a non-canonical function of intracellular CD59 in insulin secretion, separate from its canonical cell-surface role in inhibition of MAC deposition.

This work was followed up by a study in which CD59 was knocked down using siRNA, and then replaced by transfection with synonymously coded cDNA constructs that escaped siRNA-mediated targeting but contained various targeted mutations. The main finding from this study concerned the GPI anchor; a version of CD59 lacking the C-terminal GPI signal peptide and attachment site rescued insulin secretion in cells lacking endogenous CD59, showing that non-GPI-anchored CD59 isoforms were involved in insulin secretion (94). When un-anchored, CD59 within the secretory pathway was retrotranslocated into the cytosol, a process dependent on recognition of the trimmed N-linked glycosylation site, and once within the cytosol this form of CD59 interacted with insulin secretion machinery. This was confirmed in β-cell clones where PIG-A was knocked out using CRISPR/Cas9: these cells did not express cell-surface CD59 but were still able to secrete insulin in response to glucose, unless CD59 expression was knocked down using siRNA, showing that secretion was reliant on non-GPI-anchored CD59 (94).

While the mechanisms of the function of CD59 in insulin granule exocytosis are still being investigated, the interactions are mediated via the specific protein domain of CD59, as removal of CD55, a similar GPI-anchored complement regulator, had no effect on insulin secretion (95), and overexpression of Thy1, another cell-surface GPI-linked protein, did not rescue insulin secretion in CD59-deficient cells (86). While results from cell lines are clear-cut, the physiological relevance is still to be confirmed. While multiple rodent models of diabetes displayed altered islet expression of CD59 (86), we have not seen significant changes in overall CD59 transcripts in human samples. CD59 is however highly expressed and abundant at the cell surface, in keeping with its important role in cellular defence against complement, and if only a small subset of total CD59 is targeted to the cytosolic environment, then even significant changes in this sub-population may not translate to noticeable changes in total CD59 transcript levels. In addition, there are no reports of diabetic phenotypes in CD59 knockout animals, although this may not have yet been studied directly. There may be compensatory mechanisms in play in mice, which have two copies of the CD59 gene with differing tissue expression patterns (96). It is interesting to note that the CD59 gene identified in guinea pigs lacks a functional domain for GPI anchorage and was not expressed at RNA levels in tested tissues (the brain, liver, lung, muscle, kidney and cervix) (97), and therefore may also have limited tissue expression and function in regulated secretion in an intracellular manner.

As for human cases, rare patients exist with germline-inherited mutations in CD59, and therefore lack functional cell-surface CD59 in all cells. These patients not only experience haemolysis, but also develop progressive childhood demyelinating neuropathy (36) that can be fully treated by clinically available complement inhibitors. So far, insulin secretion deficiencies have not been described in these patients, but CD59 cDNA containing one of the reported mutations, C89Y, did rescue insulin secretion, despite this mutant not reaching the cell surface, and it would therefore appear that the intracellular and cell-surface functions of CD59 have separate structural requirements.

## C4BP

Islet amyloid polypeptide, IAPP, is a hormone peptide that is co-secreted with insulin from β-cells. IAPP also forms amyloid deposits in the pancreas, with increased amyloid deposition in diabetes, correlating with β-cell loss (98). We discovered previously that complement, which is known to be involved in clearance of dead cells and protein aggregates, also interacts with amyloid deposits (99, 100). In particular, complement inhibitor C4b-binding protein (C4BP) has a role in regulating complement activation so that IAPP deposits are detected and cleared, without causing excessive complement-mediated inflammation (100). IAPP can however be directly cytotoxic, as its oligomers/multimers interact with and disrupt cell membranes (101). We also showed that C4BP binds to IAPP oligomers, and blocks their cytotoxic activity, protecting β-cells (102). IAPP oligomers can also activate the inflammasome, becoming internalised by myeloid cells and disrupting lysosomal integrity (103), leading to NLRP3 activation and release of the pro-inflammatory cytokine IL-1β, which contributes to islet inflammation and β-cell dysfunction (104). C4BP, which is expressed in delta-cells of the pancreatic islet, also inhibited IAPP-dependent inflammasome activation, with a resultant rescue of β-cell function and insulin secretion (105). C4BP therefore acts as both an inhibitor of complement-dependent activation, but also has a non-canonical role as an inhibitor of IAPP-mediated cytotoxicity and inflammation independent of its complement-regulatory function, therefore acting at several levels to protect homeostasis and function of the pancreatic islet (106).

## Conclusions

It is intriguing to note that as well as the evidence for the involvement of complement in influencing cellular metabolism in mammals, it has recently been shown that even insects lacking C3-related thioester-containing proteins have altered carbohydrate and triglyceride levels (107), suggesting an evolutionarily ancient link between complement and metabolism (18). Diabetes is a complex disease, involving interactions of many tissues and cell types at different anatomical sites within the body. Apart from the classification into T1D and T2D, it is now apparent that T2D can be subdivided into further subtypes, defined by different clinical features and with differing genetic associations. The heterogenous nature of the disease may have previously obscured roles of distinct inflammatory pathways, which may play different roles in distinct diabetes subtypes. The identification of these novel subtypes is therefore a first step towards a ‘personalised medicine’ approach, where specific pathways can be targeted in specific patients.

**Fig. 2:**
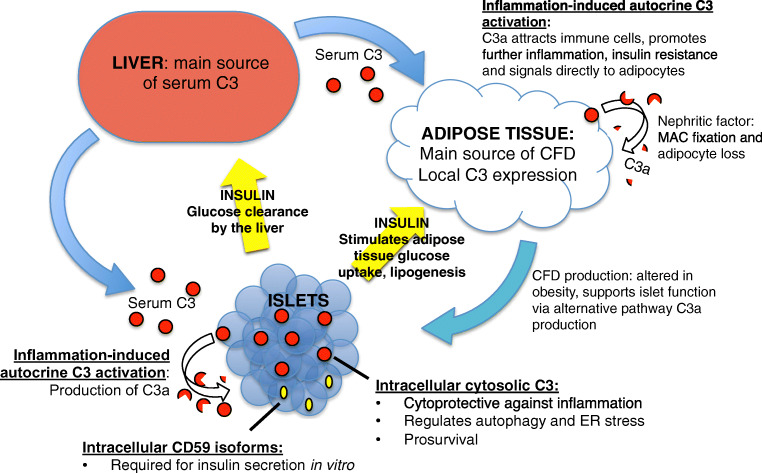
Interactions of complement with metabolic tissues. Serum complement proteins are derived mainly from the liver with the exception of FD, which is expressed primarily in adipocytes and is altered in obesity. Local C3 expression is also found at various anatomical sites. In particular, C3 is highly expressed in human islets. Both circulating levels of C3 and local islet C3 expression are upregulated during T2D. Notably, C3a has been shown to have direct effects on both adipocytes and β-cells, while intracellular isoforms of both C3 and CD59 play homeostatic roles in β-cell survival and function

Similarly, the complement system has a complex role in the disease, playing opposing beneficial and detrimental roles in different tissues (Fig. 2). Inflammation plays a direct role in inducing both insulin resistance and β-cell dysfunction, and there is a clear role for the canonical functions of complement in recruiting pro-inflammatory leukocytes to adipose tissue, as well as in causing direct pathology in the clinical complications of chronic diabetes. However, there is increasing evidence for beneficial local roles of complement in tissue homeostasis of both adipose tissue and pancreatic islets, in particular for roles of both the alternative pathway, and C3a, as well as for intracellular complement, in the case of intracellular isoforms of both CD59 and C3. With FD being primarily produced in adipose tissue, and C3a seeming to act on islets to improve function and survival, the alternative pathway of complement also seems to present a novel means of communication between these different tissues, although how this may be targeted specifically to β-cells has not been investigated. The nature of complement as a ‘two-edged sword’, capable of both negatively and positively influencing whole-body metabolism by differing activity in different tissues, calls for further work to understand the details of these relative contributions. In particular, novel tissue-specific knockout mice are required to understand relative contributions of circulating and locally produced complement factors, as well as relative roles of intra- and extracellular proteins. Inflammatory pathways are now being carefully considered as therapeutic targets in diabetes and metabolic disease (108), and inhibitors of complement are finding their way towards the clinic (109). Better understanding of the mechanisms and outcomes of complement pathways, and their interplay with metabolism, will path the way to improved personal medicine and tailored treatments of metabolic disturbances.
